# Basophilic green fluorescent carbon nanoparticles derived from benzoxazine for the detection of Cr(vi) in a strongly alkaline environment†

**DOI:** 10.1039/c7ra10814a

**Published:** 2018-02-15

**Authors:** Bin Fang, Ping Wang, Yujia Zhu, Caoyu Wang, Geng Zhang, Xinsheng Zheng, Cong Ding, Jiangjiang Gu, Feifei Cao

**Affiliations:** College of Science, Huazhong Agricultural University Wuhan 430070 P. R. China jiangjianggu@mail.hzau.edu.cn caofeifei@mail.hzau.edu.cn; Departments of Radiation Oncology, State Key Laboratory of Oncology in South China, Collaborative Innovation Center for Cancer Medicine, Sun Yat-sen University Cancer Center Guangzhou 510060 P. R. China

## Abstract

Fluorescent probes for heavy or transition metal ions in extreme environments are crucially important for practical use. In this work, basophilic green fluorescent carbon nanoparticles (G-CNPs) were synthesized by one-pot hydrothermal treatment of benzoxazine in NaOH aqueous solution. These G-CNPs showed favorable dispersibility in strongly alkaline conditions due to the abundant functional groups on their surface. Based on their good photoluminescence properties and excellent stability, the G-CNPs could be used to detect Cr(vi) in a strongly alkaline environment (pH = 14) through a fluorescence quenching effect. This detection process was achieved selectively among 17 anions within 30 seconds and the limitation was 0.58 μM (S/N = 3). It was revealed that the fluorescence turn-off process was caused by the inner filter effect (IFE) of Cr(vi). This study developed efficient fluorescence sensors based on fluorescent carbon nanoparticles, which could be used in strongly alkaline environments.

## Abstract 2

Basophilic green fluorescent carbon nanoparticles (G-CNPs) were prepared from benzoxazine for the selective detection of Cr(vi) in strongly alkaline environments.

## Graphical abstract

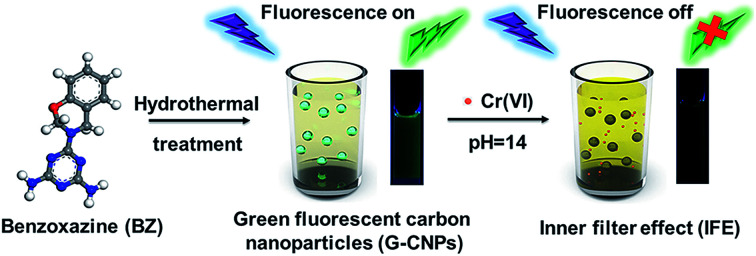

## 1. Introduction

The detection of heavy metal ions in the environment has a great importance for the prevention of pollution and the protection of human health.^1–3^ It is reported that fluorescence analysis is an efficient method to achieve this purpose thanks to its convenient operation, high sensitivity and good visualization.^4,5^ Hence, fluorescent materials play a key role in excellent fluorescence strategies. Traditional fluorescent materials contained organic dyes, rare earth complexes, conjugated polymers and semiconductor quantum dots (QDs), while emerging fluorescent carbon nanoparticles (CNPs) including carbon dots (CDs) have attracted more and more attention in recent years.^6–12^

CNPs have been studied in depth since they were first discovered in 2004.^13^ They are widely used due to their excellent fluorescence, environmental friendness, easy synthesis and low-cost.^14–18^ In particular, CNPs have already been applied as fluorescent probes to detect metal ions. For instance, Zheng *et al.* used CNPs as the fluorescent turn-off probe to detect Cr(vi) based on the inner filter effect (IFE).^19^ Algarra and co-workers demonstrated that luminescent CNPs which were synthesized from lactose and modified with mercaptosuccinic acid could be used for efficient quantification of Ag(i) though fluorescence quenching effect.^20^ Kar's group produced hollow fluorescent CNPs and showed that they had selective response for Fe(iii) *via* a “turn-off” mechanism.^21^ Other metal ions such as Cu(ii),^22,23^ Hg(ii),^24–26^ Pd(ii),^27^ Au(iii)^28^ and Al(iii)^29^ were also evaluated by the fluorescent sensors based on CNPs. However, most of these work focused on the detection in mild conditions with pH at 2–12. In actual situations, extreme treatment of samples is a necessary procedure for the accurate quantitative evaluation of metal ions and interference reduction.^30,31^ Thus, the carbon nanoparticle probes for certain metal ions in extreme conditions are highly demanded. Tang *et al.* showed S-doped CNPs could achieve the detection of Fe(iii) in strong acid conditions (pH = 0).^32^ However, fluorescent sensors based on CNPs for metal ion detection in strongly alkaline conditions are still a huge challenge.

In this study, benzoxazine (BZ) was used as the novel raw material to prepare green fluorescent CNPs (G-CNPs) *via* hydrothermal treatment in NaOH aqueous solution. The obtained G-CNPs demonstrated good solubility in the basic medium and showed green light under UV radiation. Based on these properties, the detection performance of G-CNPs towards various anions in the strongly alkaline environment (pH = 14) was investigated and the G-CNPs showed selective and rapid response to Cr(vi) with a low limitation of 0.58 μM (S/N = 3). Then the selective detection performance of G-CNPs was proved to result from the IFE of Cr(vi).

## 2. Experimental

### 2.1. Chemicals

Melamine, phenol, formaldehyde solution (37–40 wt%), methanol, HCl, NaOH, NaCl, K_2_CO_3_, KNO_3_, NaNO_2_, Na_2_SO_4_, Na_2_SO_3_, Na_2_S, Na_2_S_2_O_3_, K_2_Cr_2_O_7_, Sodium aceticum (NaAc), KF, KCl, KBr, KI, KMnO_4_, K_3_Fe(CN)_6_, K_2_FeO_4_ and Zn(OH)_2_ were provided by Sinopharm Chemical Reagent Co., Ltd. (Shanghai, China). Quinine sulfate was purchased from Adamas-beta Inc. (Shanghai, China). All chemicals were of analytical grade and used directly without further purification. Ultrapure water (18.2 MΩ cm^−1^) used in this study was obtained from a Millipore water purification system.

### 2.2. Synthesis of BZ

In a typical process, 6.4 g melamine, 4.5 mL of phenol and 25 mL of aqueous formaldehyde (37–40 wt%) were introduced into 100 mL methanol in a 250 mL round-bottom flask under vigorous stirring at 80 °C for 6 h. Afterwards, a decompressing distillation was used to remove methanol from the reaction mixture at 55 °C. Then the mixture was dissolved in chloroform and washed by sodium hydroxide aqueous solution to remove the unreacted phenol. Finally, the chloroform was removed and the obtained liquid was of low-viscosity, transparent and colorless, indicating that BZ was successfully synthesized.

### 2.3. Preparation and purification of G-CNPs

0.3 g of BZ and 30 mL of 0.5 M NaOH aqueous solution were mixed and transferred into a 50 mL Teflon equipped stainless steel autoclave, which was heated at 200 °C for 6 h. After the autoclave was cooled down to room temperature, the obtained brownish yellow solution was filtered by 220 nm membrane. When neutralized to pH = 7.0 by adding the diluted HCl, the G-CNPs precipitated from solution immediately. This suspension was centrifuged with 10 000 rpm for 5 min to precipitate out G-CNPs. Then the product was washed by water and centrifuged for 3 times. Finally, the wet product was freeze-dried to obtain G-CNPs for the further use.

### 2.4. Characterization

The transmission electron microscopy (TEM) image was taken by a H-7650 electron microscope (Hitachi, Japan) at an accelerating voltage of 80 kV. The high resolution transmission electron microscopy (HRTEM) image was observed with a Talos F200C electron microscope (FEI, USA) at an accelerating voltage of 200 kV. The scanning electron microscopy (SEM) image was obtained using a SU8010 electron microscope (Hitachi, Japan). The elemental analysis was measured with a vario EL III elemental analyzer (Elementar, Germany). The X-ray diffraction (XRD) pattern was performed using a Rigaku D/max-2500 diffractometer with a filtered Cu Kα radiation. Fourier transform infrared (FT-IR) spectroscopy were conducted on a NEXUS 670 FTIR spectrometer, ranging from 400 to 4000 cm^−1^. The X-ray photoelectron spectroscopy (XPS) result of the sample was collected using an ESCALab 250Xi XPS instrument (Thermo Scientific). Ultraviolet-visible (UV-vis) absorption spectra were recorded by a Shimadzu 2450 UV-vis spectrophotometer (Shimadzu, Japan). All fluorescence spectra were measured by a RF-5301PC fluorescence spectrometer (Shimadzu, Japan) with 5 nm slit width for the excitation and emission.

### 2.5. Ion sensing capacity measurements

The detection of Cr(vi) was performed in a strongly alkaline solution (pH = 14). Typically, 300 μL of G-CNPs (5 mg mL^−1^, pH = 14) were dispersed into 2.4 mL of 1 M NaOH aqueous solution, then 300 μL of Cr(vi) solution (pH = 14) was added and the final concentration of Cr(vi) was in the range of 0–500 μM. After the solution was incubated for 30 s, the emission spectra were recorded at 380 nm excitation. For comparison, the same procedure was also performed for other ions at the final concentration of 200 μM.

### 2.6. Detection of Cr(vi) in real samples

The tap water and lake water samples were collected from our laboratory and the south lake (Wuhan, China), respectively. All the samples were filtered through 0.22 μM membranes to remove the large suspended particles and their pH levels were regulated to 14. Then 2.4 mL of water sample, 300 μL of Cr(vi) solution (pH = 14) and 300 μL of G-CNPs (5 mg mL^−1^, pH = 14) were mixed and recorded with the fluorescence spectrometer.

## 3. Results & discussion

**Scheme 1:**
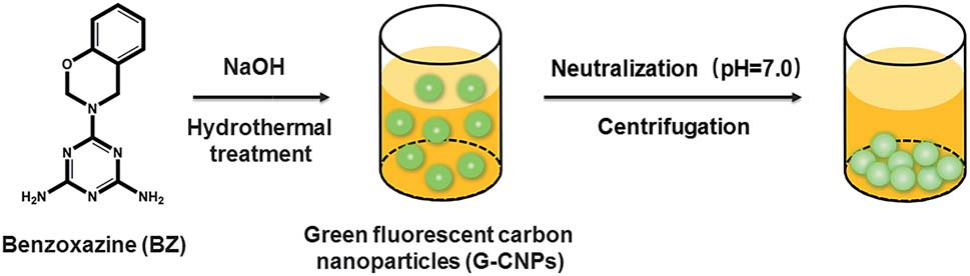
A schematic illustration of the formation and purification of G-CNPs by hydrothermal treatment of BZ.

As the raw material to prepare G-CNPs, BZ was synthesized according to the reported literature,^33^ which was shown in Fig. S1 (ESI†). BZ was formed from melamine, phenol and formaldehyde though Mannich reaction. The FT-IR result in Fig. S2 (ESI†) demonstrates two absorbance peaks at 1498 and 904 cm^−1^, which are attributed to the in-plant C–C stretching and the out-of-plane C–H deformation of substituted benzene ring in the benzoxazine structure. The peaks at 1565 and 3340 cm^−1^ are corresponding to C=C in the aromatic ring and N–H in the primary amine, respectively. The characteristic bands at 1178 and 1070 cm^−1^ can be assigned successively to asymmetric stretching of C–N–C and symmetric stretching of C–O–C of the oxazine ring, which confirms the successful synthesis of BZ. The formation of G-CNPs from BZ is schematically shown in Scheme 1. Briefly, BZ and NaOH aqueous solution were added in a Teflon equipped stainless steel autoclave and heated to 200 °C for 6 hours. During the hydrothermal process, the undissolved BZ could be pyrolyzed and carbonized to form G-CNPs. Then the clear brownish yellow solution was obtained with little precipitate. After the neutralization with HCl, G-CNPs aggregated and the precipitate was easily separated through centrifugation. This treatment did not need dialysis or column chromatography like other CNPs, which is considered as a simple and efficient way to fabricate G-CNPs. For comparison, hydrothermal treatment of BZ under a neutral condition was also done and lots of unreacted BZ remained (data was not shown). It is inferred that NaOH could promote the pyrolysis of BZ and the formation of G-CNPs.

**Fig. 1:**
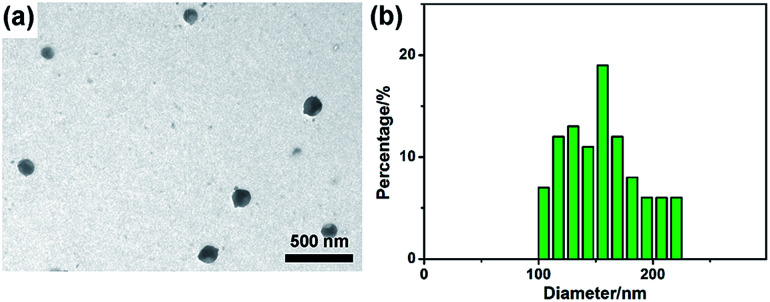
TEM image (a) and the corresponding particle size distribution histogram (b) of G-CNPs.

To investigate the morphology of obtained G-CNPs, a TEM image was captured and shown in Fig. 1a. It is clear that the final product consists of well separated spherical nanoparticles with the size of around 150 nm. The corresponding particle size distribution histogram in Fig. 1b shows that the diameter of G-CNPs ranges from 100 to 220 nm with a mode around 156 nm. The HRTEM image in Fig. S3 (ESI†) reveals the amorphous construction of G-CNPs without any clear lattice fringes. The G-CNPs were also characterized by SEM. As shown in Fig. S4a (ESI†), spherical nanoparticles with mean size of 159 nm (Fig. S4b, ESI†) are observed, which is consistent with the TEM result. The XRD patterns as shown in Fig. S5 (ESI†) further identify the crystallinity of the G-CNPs. The broad diffraction peak (2*θ*) centered at 23.1° clearly reveals that the G-CNPs possess an amorphous carbon phase, which conforms to the results of HRTEM.

**Fig. 2:**
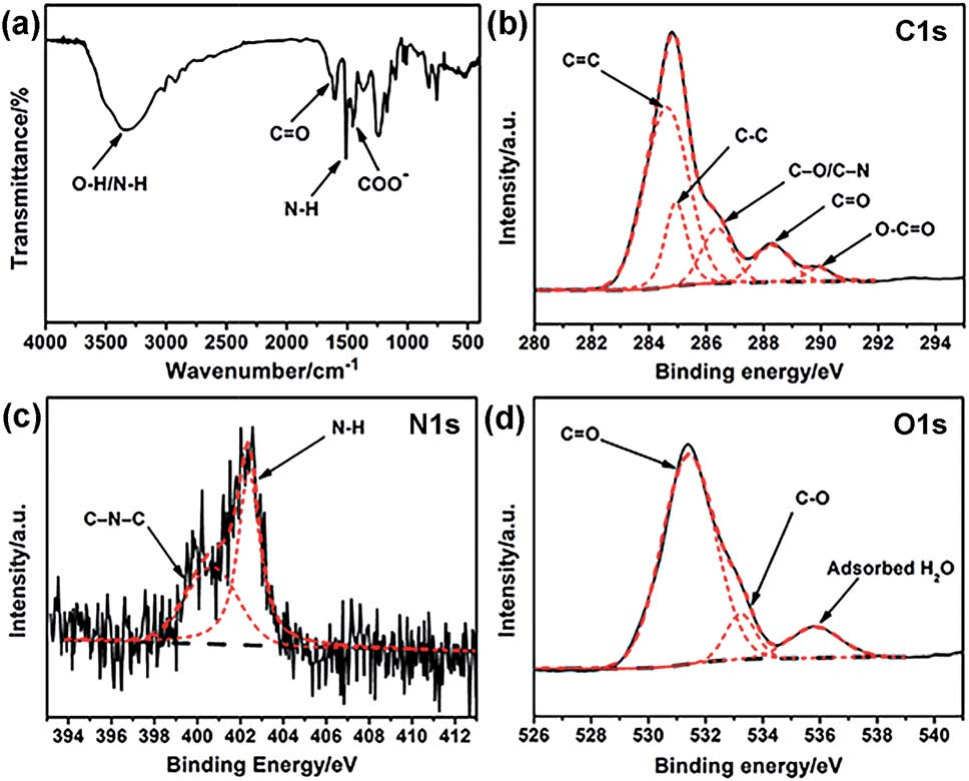
(a) FT-IR spectrum of G-CNPs. High resolution (b) C 1s, (c) N 1s and (d) O 1s XPS spectra of G-CNPs. Black solid line: raw, black dash line: background, red dash line: fitting.

Elemental analysis was carried out to analyze the elemental composition of the obtained G-CNPs and the result is shown in Table S1 (ESI†). The contents of C, H and N in G-CNPs are 54.78%, 4.48% and 1.04%, indicating that the G-CNPs is doped by nitrogen successfully. To characterize the functional groups of G-CNPs, FT-IR spectroscopy was performed and the result is shown in Fig. 2a. The absorbance peak of C–O–C symmetric stretching in the oxazine ring disappeared, indicating that the decomposition of the oxazine ring happened during the hydrothermal process. The new absorption peak at around 1650 cm^−1^ is due to C=O stretching vibration in carboxy groups. Furthermore, the peak at about 1451 cm^−1^ is contributed to COO^−^, which causes the excellent dispersibility of G-CNPs in the strongly alkaline solution. The presence of C=O and COO^−^ are caused by the pyrolysis, oxidation and polymerization of benzoxazine in the hydrothermal process.^15,34–36^ The peak at 1510 cm^−1^ is attributed to the bending vibrations of N–H groups, which originates from the benzoxazine.^36^ The broad absorption peaks at 3510 and 3343 cm^−1^ can be assigned to O–H and N–H stretching vibrations, respectively. Zeta potential of G-CNPs is −16.7 ± 3.3 mV, suggesting that carboxyl groups were distributed on the surface of G-CNPs.

XPS was used to identify the surface element states of the obtained G-CNPs. As shown in Fig. 2b, the five peaks at 284.4, 284.9, 286.6, 288.2 and 289.9 eV in the C 1s spectrum can be assigned to C=C, C–C, C–O/C–N, C=O and O–C=O groups, respectively.^22,34,37–39^ N 1s spectrum in Fig. 2c exhibites two peaks at 400.4 and 402.4 eV, which are assignable to C–N–C and N–H, respectively. The O 1s spectrum (Fig. 2d) can be deconvoluted into three peaks at 531.4, 533.2 and 535.8 eV, which are ascribed to C=O, C–O and absorbed H_2_O, respectively. The XPS results also confirm that large number of hydrophilic carboxyl groups exist on the surface of G-CNPs.

**Fig. 3:**
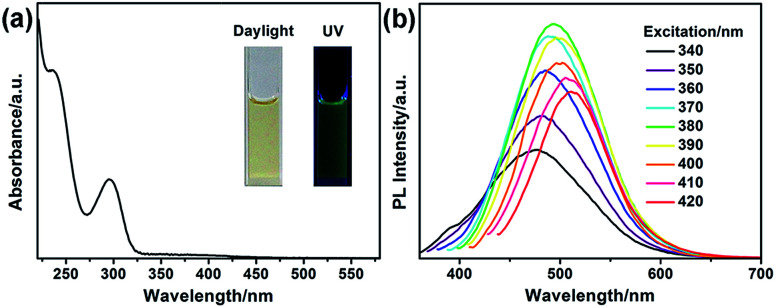
(a) UV-vis absorption spectrum of G-CNPs, inset: photographs of G-CNPs in an aqueous solution (pH = 14) under daylight and UV irradiation. (b) PL spectra of G-CNPs at different excitation wavelengths.

The optical properties of G-CNPs were described by UV-vis absorption and photoluminescence (PL) spectra. As shown in the Fig. 3a inset, the solution of the G-CNPs (pH = 14) was yellowish and transparent in daylight and demonstrated obvious green fluorescence under UV irradiation (365 nm), suggesting the excellent dispersibility of G-CNPs in the strong alkaline environment. The UV-vis absorption spectrum of as-prepared G-CNPs (Fig. 3a) displayed two peaks at 236 and 296 nm emerging from the π → π* and n → π* transitions as previous reports.^34,40^ The PL spectra of G-CNPs with various excitation wavelengths are shown in Fig. 3b. When the excitation wavelength increases from 340 to 420 nm, the wavelength of the maximum emission shifts from 478 to 511 nm, which is more obvious in Fig. S6 (ESI†). This phenomenon of excitation-dependent red shift in PL spectra is similar to other reported CNPs or CDs, which is resulted from the complex surface defects and the polydispersity of G-CNPs.^41–45^ The PL intensity of the emission peak approaches maximum at 495 nm under 380 nm excitation, which is suitable to be set as the recording condition for the analysis.

**Fig. 4:**
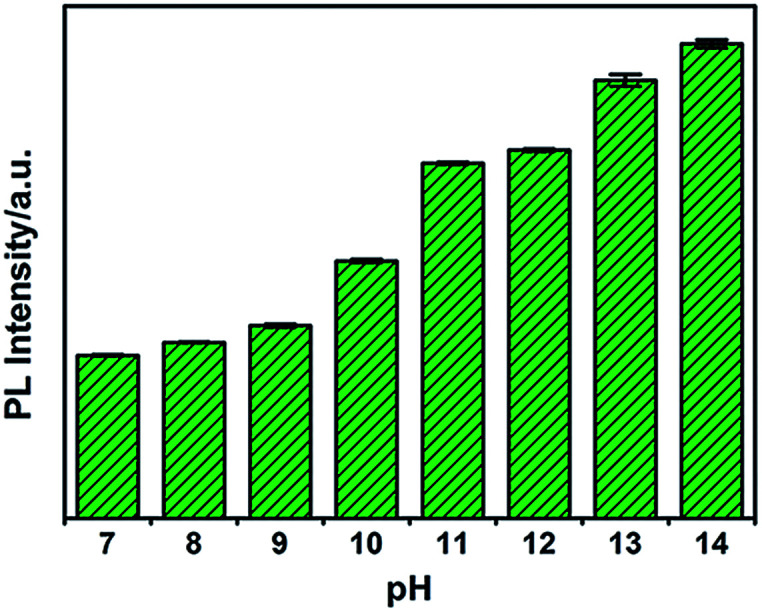
pH effect on PL emission intensity (495 nm) of G-CNPs under 380 nm excitation.

To investigate the influence of pH on the fluorescence of G-CNPs, the PL intensity (495 nm) of G-CNPs at various pH values (7–14) was recorded under 380 nm excitation. As illustrated in Fig. 4, the PL intensity increased with the alteration of pH. As mentioned, the G-CNPs had a large number of carboxyl groups on their surface, and the carboxyl groups exist as carboxylate at pH = 14. As the pH decreases, more and more carboxylate groups protonate and the repulsive force between G-CNPs became weaker, which causes the aggregation of particles and the reduction of fluorescence. The G-CNPs process the strongest fluorescence at pH = 14, which indicates that G-CNPs may have potential applications in the strongly alkaline environment.

**Fig. 5:**
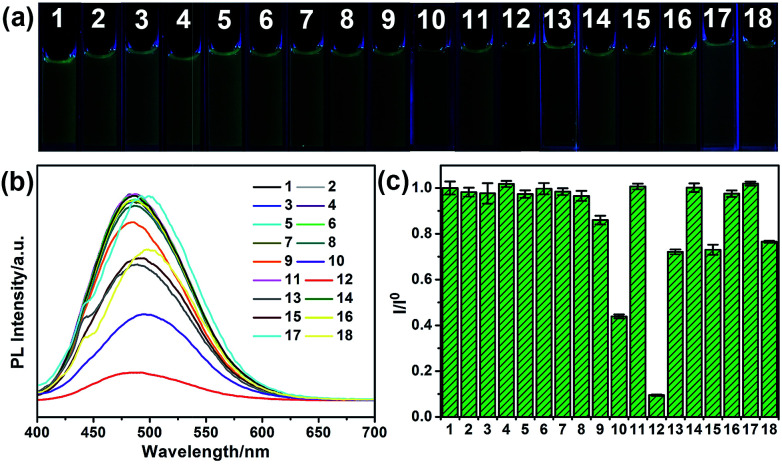
Selectivity of G-CNPs with different ions. (a) The optical photograph of G-CNPs dispersed in NaOH (pH = 14) with different ions (200 μM) under UV irradiation. (b) Corresponding PL spectra at 380 nm excitation. (c) The relationship between (*I*/*I*^0^) and ions. *I* and *I*^0^ were the PL intensities (495 nm) of G-CNPs at 380 nm excitation in the presence and absence of different ions, respectively. 1: G-CNPs, 2: CH_3_COO^−^, 3: F^−^, 4: Cl^−^, 5: Br^−^, 6: I^−^, 7: NO_2_^−^, 8: NO_3_^−^, 9: ClO^−^, 10: MnO_4_^−^, 11: CO_3_^2−^, 12: Cr(vi), 13: FeO_4_^2−^, 14: SO_3_^2−^, 15: S_2_O_8_^2−^, 16: SO_4_^2−^, 17: ZnO_2_^2−^, 18: Fe(CN)_6_^3−^.

To explore the selectivity of G-CNPs as fluorescent probes for the ion detection in the strong alkaline condition, different ions were introduced into the G-CNPs at pH 14. Seventeen anions including CH_3_COO^−^, F^−^, Cl^−^, Br^−^, I^−^, NO_2_^−^, NO_3_^−^, ClO^−^, MnO_4_^−^, CO_3_^2−^, CrO_4_^2−^, FeO_4_^2−^, SO_3_^2−^, S_2_O_8_^2−^, SO_4_^2−^, ZnO_2_^2−^ (obtained by adding zinc hydroxide into a strong alkaline solution) and Fe(CN)_6_^3−^ at the concentration of 200 μM were added into G-CNPs (0.5 mg mL^−1^) dispersed in NaOH aqueous solution. As shown in Fig. 5a, the optical photographs of the mixed solutions under UV irradiation (365 nm) show that Cr(vi) could quench the green fluorescence of G-CNPs clearly compared with other anions. Furthermore, the fluorescence emission spectra were measured at 380 nm excitation (Fig. 5b) and the corresponding *I*/*I*^0^ (the ratio of PL intensities of G-CNPs in the presence and absence of anions) is demonstrated in Fig. 5c. Most of the added anions did not cause obvious fluorescence change of the G-CNPs, while ClO^−^, MnO_4_^−^, FeO_4_^2−^, S_2_O_8_^2−^ and Fe(CN)_6_^3−^ could partially quench the fluorescence of G-CNPs. G-CNPs with Cr(vi) system had the highest fluorescence quenching efficiency as high as 90.4%, which suggests that the G-CNPs have a selective response to Cr(vi).

**Fig. 6:**
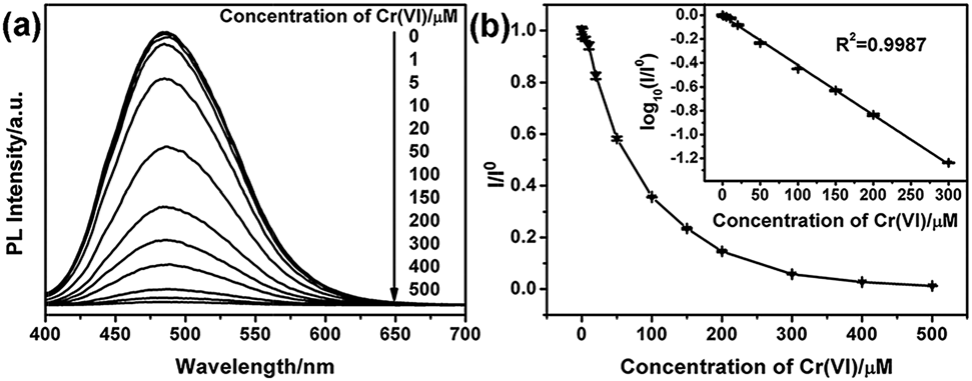
(a) PL emission spectra of G-CNPs dispersed in NaOH (pH = 14) with different concentrations of Cr(vi) (0–500 μM). (b) The relationship between *I*/*I*^0^ and Cr(vi) concentration from 0 to 500 μM, inset: the relationship between log_10_(*I*/*I*^0^) and Cr(vi) concentration from 0 to 300 μM. *I* and *I*^0^ were the PL intensities (495 nm) of G-CNPs at 380 nm excitation in the presence and absence of Cr(vi), respectively.

To analyze the detection limitation of G-CNPs towards Cr(vi), different concentrations of Cr(vi) (0–500 μM) were added into G-CNPs and the fluorescence spectra were measured at 380 nm excitation. As shown in Fig. 6a, the PL intensity at 495 nm decreases gradually as the concentration of Cr(vi) increases, which has the same tendency as the relationship between *I*/*I*^0^ and the concentration of Cr(vi) presented in Fig. 6b. A good fitted regression line (*R*^2^ = 0.9987) is observed between log_10_(*I*/*I*^0^) and the concentration of Cr(vi) in the range of 1 to 300 μM (Fig. 6b inset), whose relationship is described in the following equation:log_10_(*I*/*I*^0^) = −0.00415*c* − 0.0038in which *c* is in the range of 1–300 μM. The detection limitation was calculated as 0.58 μM (S/N = 3), which is with or lower than the recent work based on CDs in mild pH (Table S2, ESI†). These results indicate that the G-CNPs could be applied to evaluate Cr(vi) quantitatively in the strong alkaline solution.

Other important properties of analytical procedure were investigated for example the response time. As demonstrated in Fig. S7 (ESI†), the time-dependent PL intensity of G-CNPs with Cr(vi) indicates that fluorescence intensity decreased quickly after the addition of Cr(vi) into G-CNPs and was stabilized within 30 s. This result shows a fast response for the detection system. The pH effect on G-CNPs for the detection of Cr(vi) is shown in Fig. S8 (ESI†). The fluorescence quenching efficiency reaches the highest at pH = 14, which indicates the optimal measurement condition was pH = 14. The G-CNPs has the lowest fluorescence quenching efficiency towards Cr(vi) at pH = 7. One possible reason is that a small number of HCrO_4_^−^ and Cr_2_O_7_^2−^ are formed in the neutral condition.^46^

To evaluate the amount of Cr(vi) in real samples and their effect on the detection of Cr(vi), tap water and lake water were tested^36,47^ and the results are shown in Table S3 (ESI†). In fact, the fluorescence spectra of tap water and lake water with G-CNPs have no obvious change compared with that of G-CNPs, indicating that the Cr(vi) concentration in these two samples is lower than the detection limit of G-CNPs. Then different concentration of Cr(vi) is added into the real water samples with G-CNPs. The recovery efficiencies of tap water and lake water are in range of 102.3–105.0% and 99.9–101.0%, respectively. And the corresponding relative standard deviations (RSD) are 1.31–2.24% and 0.71–1.20%, respectively. These results reveal that the G-CNPs can be used for the determination of Cr(vi) in some practical water samples.

**Fig. 7:**
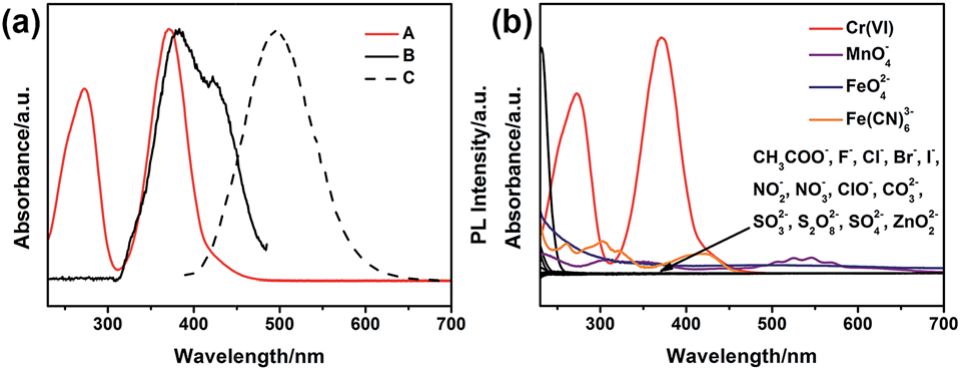
(a) UV-vis absorption spectrum of Cr(vi) (A), the maximum fluorescence excitation (B) and emission (C) spectra of the G-CNPs at pH = 14. (b) UV-vis absorption spectra of various ions (200 μM) at pH = 14.

The inner filter effect (IFE) refers to the absorption of the excitation and/or emission of fluorescent material by absorbers in the detection system, and it needs that the absorption band of the absorbers possesses a complementary overlap with the excitation and/or emission spectra of the fluorescent material.^47–51^ To confirm the mechanism of the selective response of G-CNPs towards Cr(vi), UV-vis absorption spectrum of Cr(vi) (actually CrO_4_^2−^), the maximum fluorescence excitation and emission spectra of the G-CNPs at pH = 14 were measured. As shown in Fig. 7a, the strong absorption band of Cr(vi) can overlap well with the excitation band of G-CNPs.^52,53^ When the excitation light reaches the solution contained G-CNPs and Cr(vi), Cr(vi) can absorb most of the light and the G-CNPs can not get enough light to excite the green fluorescence. Therefore, Cr(vi) can shield the excitation light for G-CNPs, which achieve the IFE. Meanwhile, Cr(vi) has no absorption peak around 495 nm, which can not cause the absorption of emission band of G-CNPs and fluorescence resonance energy transfer (FRET) between Cr(vi) and G-CNPs.^49^ To confirm that the Cr(vi) has the specific absorption band, the UV-vis absorption spectra of various ions (200 μM) at pH = 14 are shown in Fig. 7b.^51,54^ The results reveal that only Cr(vi) has the strong absorption around 380 nm, and other ions could not result in IFE. As a consequence, G-CNPs can be applied as the specific fluorescence turn-off probe to detect Cr(vi) in the strongly alkaline medium, which is proposed as the IFE.

## 4. Conclusions

In summary, novel basophilic G-CNPs derived from benzoxazine were produced *via* hydrothermal process in the presence of NaOH. They had the size of about 150 nm and exhibited obvious green fluorescence under UV excitation. Due to the large number of hydrophilic carboxyl groups on the surface of G-CNPs, they had an excellent solubility in strong alkaline environment. Thus, the G-CNPs could applied as efficient and rapid fluorescence turn-off sensors for the detection of Cr(vi) at pH = 14. This fluorescence quenching response was selective among 17 anions with the limitation as low as 0.58 μM in the case of Cr(vi), which was attributed to the IFE. This successful strategy would have important inspiration for the analysis in extreme conditions.

## Conflicts of interest

There are no conflicts to declare.

## Supplementary Material

RA-008-C7RA10814A-s001
