# Supplementary material for: Basophilic green fluorescent carbon nanoparticles derived from benzoxazine for the detection of Cr(vi) in a strongly alkaline environment
Source: RSC Adv. 2018 Feb 15;8(14):7377–82. doi: 10.1039/c7ra10814a (PMC9078398; doi:10.1039/c7ra10814a)
Supplement: RA-008-C7RA10814A-s001 [file RA-008-C7RA10814A-s001.pdf]

Electronic Supplementary Material (ESI) for RSC Advances.
This journal is © The Royal Society of Chemistry 2018

Electronic Supplementary Information

Basophilic green fluorescent carbon nanoparticles derived from benzoxazine for the detection of Cr(VI) in a strong alkaline environment

Bin Fang,[a] Ping Wang,[a] Yujia Zhu,[b] Caoyu Wang,[a] Geng Zhang,[a] Xinsheng Zheng,[a] Cong Ding,[a] Jiangjiang Gu*[a] and Feifei Cao*[a]

[a] College of Science, Huazhong Agricultural University, Wuhan 430070, P. R. China.

[b] Departments of Radiation Oncology, State Key Laboratory of Oncology in South China, Collaborative Innovation Center for Cancer Medicine, Sun Yat-sen University Cancer Center, Guangzhou, 510060, P. R. China

Table S1. Elemental analysis result of the G-CNPs.

Sample	C (%)	H (%)	N (%)
G-CNPs	54.78	4.48	1.04

Table S2. Comparison of different CNPs and CDs for Cr(VI) detection.

Material	pH value	Linear range (μM)	Limit of detection (μM)	Reference
N, S co-doped CDs	3, 7.4, 12	1-80	0.86	[S1]
N, S-co-doped CDs	3	1-40	0.52	[S2]
CDs	5	2–180	2.10	[S3]
Cobalt(II)-doped CDs	—	5-125	1.17	[S4]
NB-CQDs	8.0	0–250	0.5	[S5]
N,S-CDs	2	1-200	0.76	[S6]
G-CNPs	14	1-300	0.58	This work

Table S3. G-CNPs for the Cr(VI) detection in water samples.

Sample	Added Cr(VI) (μM)	Found Cr(VI) (μM)	Recovery (%)	RSD (%)
Tap water	0	Not detected	-	-
	10	10.23	102.3	1.31
	100	105.03	105.0	2.24
Lake water	0	Not detected	-	-
	10	10.10	101.0	1.20
	100	99.90	99.9	0.71

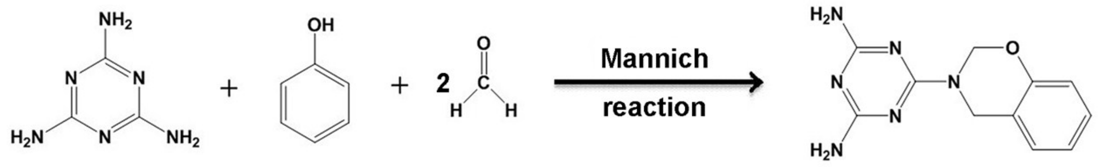

Fig. S1. The synthetic procedure of BZ.

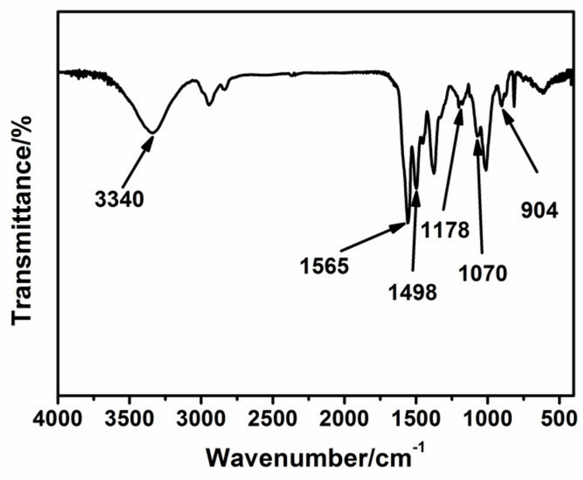

Fig. S2. FT-IR spectrum of BZ.

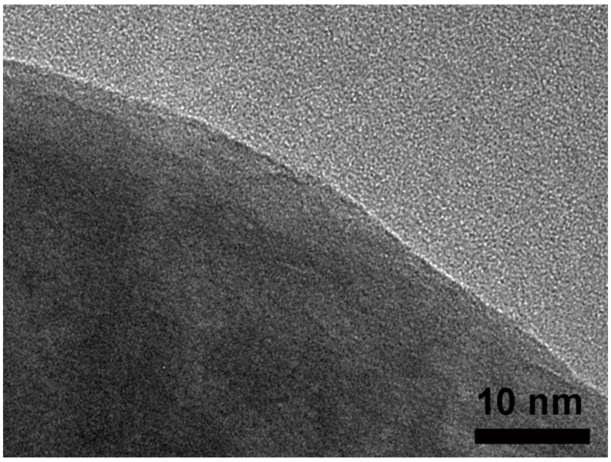

Fig. S3. HRTEM image of an individual G-CNP.

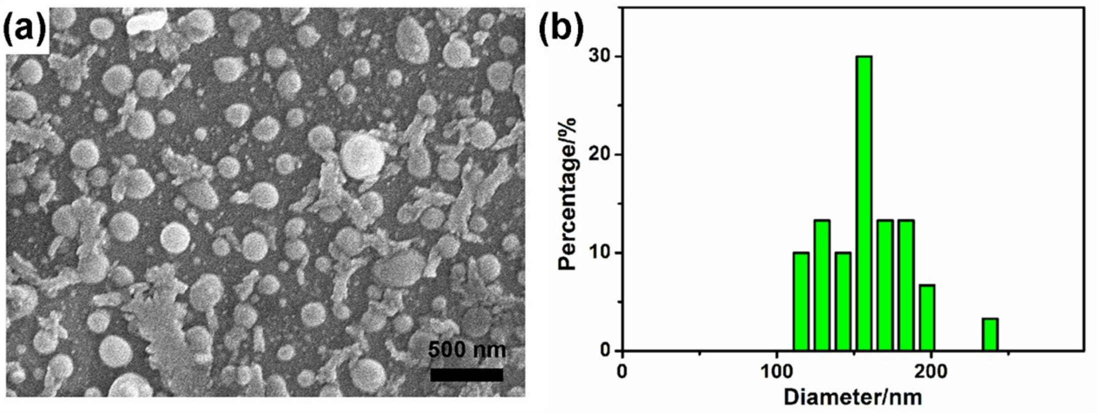

Fig. S4. SEM image (a) and the corresponding particle size distribution histogram (b) of G-CNPs.

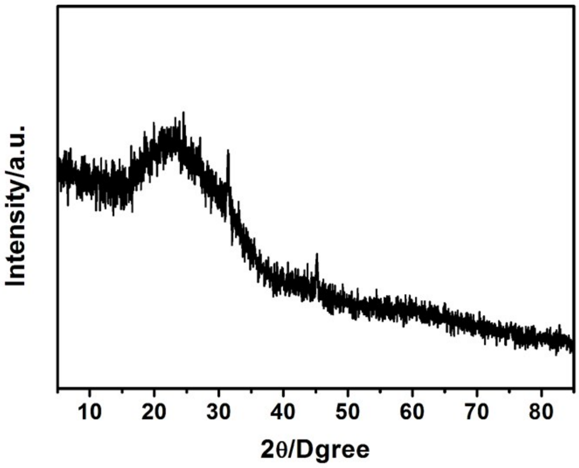

Fig. S5. XRD pattern of G-CNPs.

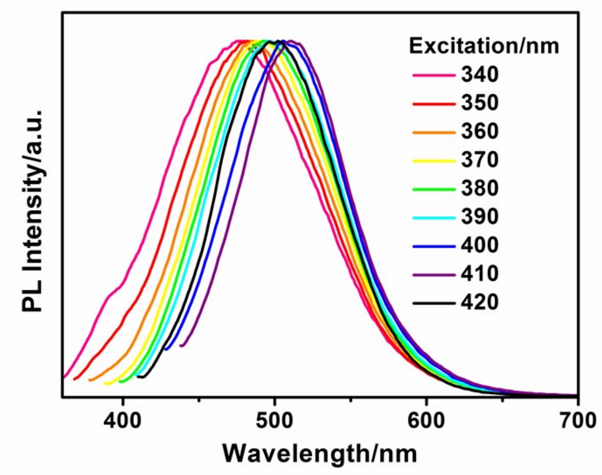

Fig. S6. Normalized PL spectra of G-CNPs at different excitation wavelengths.

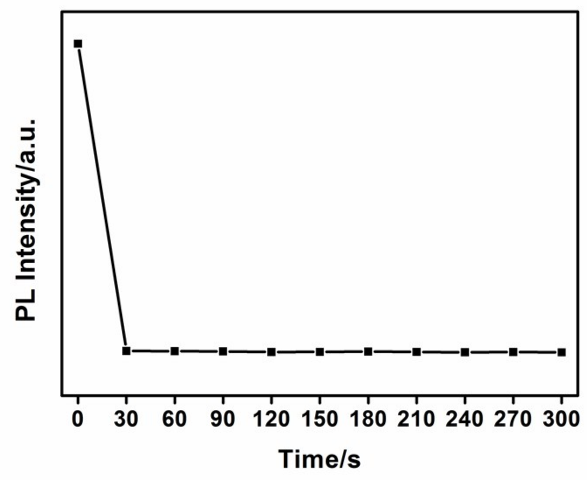

Fig. S7. Time-dependent PL intensity of G-CNPs with Cr (VI) in NaOH (pH=14).

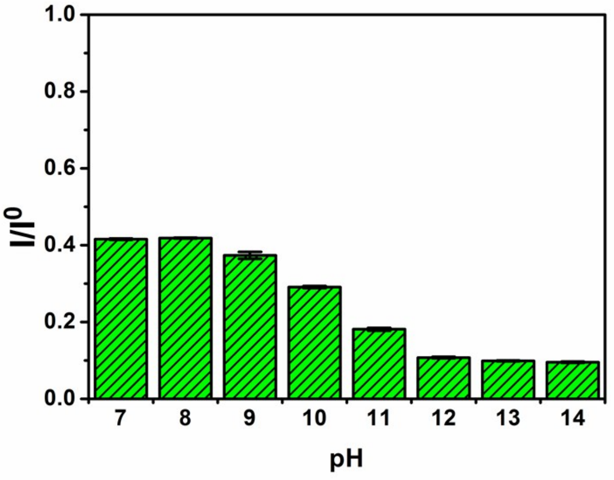

Fig. S8. pH effect on G-CNPs for the detection of Cr (VI). I and I^0 were the PL

intensities (495nm) of G-CNPs at 380 nm excitation in the presence and absence of 200 μM Cr(VI), respectively.

References

S1 J. Chen, J. Liu, J. Li, L. Xu and Y. Qiao, *J. Colloid Interf. Sci.*, 2017, **485**, 167–174.

S2 J. Shen, S. Shang, X. Chen, D. Wang and Y. Cai, *Sens. Actuators B: Chem.*, 2017, **248**, 92–100.

S3 C. Li, W. Liu, X. Sun, W. Pan and J. Wang, *Sens. Actuators B: Chem.*, 2017, **252**, 544–553.

S4 H.-Y. Zhang, Y. Wang, S. Xiao, H. Wang, J.-H. Wang and L. Feng, *Biosens. Bioelectron.*, 2017, **87**, 46–52.

S5 Y. Guo, Y. Chen, F. Cao, L. Wang, Z. Wang and Y. Leng, *RSC Adv.*, 2017, **7**, 48386–48393.

S6 J. Shen, T. Zhang, Y. Cai, X. Chen, S. Shang and J. Li, *New J. Chem.*, 2017, **41**, 11125—11137.
